# Wearing Lower-Body Compression Tights to Bed After Cycling Exercise Does Not Affect Subsequent Sleep in Healthy Male Adults

**DOI:** 10.3390/s26051625

**Published:** 2026-03-05

**Authors:** Charli Sargent, Shona L. Halson, Matthew Morrison, Carissa L. Gardiner, Dean J. Miller, Bree L. Elliott, Katrina Nguyen, James R. Broatch, Jonathon Weakley, Gregory D. Roach

**Affiliations:** 1 Appleton Institute for Behavioural Science, Central Queensland University, Goodwood 5034, Australia; d.j.miller@cqu.edu.au (D.J.M.); greg.roach@cqu.edu.au (G.D.R.); 2 S.P.O.R.T. Research Cluster, Central Queensland University, Rockhampton 4700, Australia; 3 Sports Performance, Recovery, Injuries, and New Technologies (SPRINT) Research Centre, School of Behavioural and Health Sciences, Australian Catholic University, Brisbane Campus (McAuley), Banyo 4014, Australia; shona.halson@acu.edu.au (S.L.H.); carissa.gardiner@acu.edu.au (C.L.G.); jonathon.weakley@acu.edu.au (J.W.); 4 Centre for Human Factors and Sociotechnical Systems, University of the Sunshine Coast, Sippy Downs 4556, Australia; mmorrison1@usc.edu.au; 5 Institute for Health and Sport, Victoria University, Melbourne 8001, Australia; james.broatch@vu.edu.au

**Keywords:** recovery, slow wave sleep, rapid eye movement sleep, polysomnography, cycle ergometer

## Abstract

**Highlights:**

**What are the main findings?**

- Wearing lower-body compression tights during sleep following a bout of moderate-intensity cycling exercise does not improve objective sleep quantity or quality.
- Wearing lower-body compression tights during sleep following a bout of moderate-intensity cycling exercise does not affect subjective sleep quality or perceptions of comfort or pain during sleep.

**What is the implication of the main finding?**

- Athletes who wear lower-body compression tights to bed for the perceived benefits of recovery from moderate-intensity exercise can do so without any undue effects on sleep.

**Abstract:**

There is some evidence to indicate that lower-body compression garments aid recovery from exercise by improving sleep quality, but this evidence is based on measures derived from self-reports and accelerometers. The aim of this study was to examine the impact of wearing lower-body compression tights to bed on sleep following a bout of exercise, using the gold standard for sleep measurement. Twelve healthy males participated in a within-subjects, counterbalanced, randomized study with two conditions: (i) Treatment—wearing compression tights to bed after exercise, and (ii) Control—not wearing compression tights to bed after exercise. In both conditions, participants completed 40 min of moderate-intensity exercise in the afternoon and had a 9 h sleep opportunity at night. Objective and subjective assessments of sleep were obtained using polysomnography and visual analogue scales, respectively. Wearing compression tights to bed did not affect the objective measures, including sleep onset latency (*p* = 0.572); sleep efficiency (*p* = 0.754); total sleep time (*p* = 0.953); amount of slow-wave sleep (*p* = 0.374); and amount of rapid eye movement sleep (*p* = 0.638). Furthermore, wearing compression tights to bed did not affect the subjective measures, including sleep quality (*p* = 0.549), comfort (*p* = 0.548), and pain (*p* = 0.838). Wearing lower-body compression tights to bed after moderate-intensity exercise does not improve the quantity or quality of sleep obtained. Athletes who choose to wear compression tights to bed for the perceived benefits for recovery after exercise can do so without any undue effects on sleep.

## 1. Introduction

Post-exercise recovery is an important component in the management of an athlete’s performance [1]. Several recovery modalities exist including, but not limited to, hydrotherapy [2], cryotherapy [3], massage [4], nutritional supplementation [5], and compression garments [6]. The primary goal of an effective recovery modality is to limit the impairment associated with the stressful components of training and competition. Poor recovery may increase susceptibility to injury or prevent an athlete from training at a required intensity or completing a required training load [1].

Compression garments are elastic, body-molded clothing that are typically worn on the lower body. There is some evidence to indicate that compression garments facilitate recovery from exercise by reducing perceptions of muscle soreness, pain, and fatigue [6,7,8,9,10] and increasing blood flow [11,12]. For this reason, many athletes not only wear the garments during and immediately following exercise but also wear the garments overnight during sleep [10,13,14,15,16,17]. Increased pain can delay sleep latency and increase the likelihood of waking during the night [18,19] and in animal models, pain and inflammation can diminish slow wave sleep [20,21]. It seems reasonable to suggest that compression garments could facilitate recovery from exercise by reducing the negative impact of muscle soreness and pain on subsequent sleep.

The effect of compression garments on different recovery outcomes has been examined, but assessments of sleep are rare and typically limited to self-reports of sleep quality, e.g., [22]. In one of the only studies to include objective measurements of sleep as an outcome, the effect of wearing full-length lower-body compression garments was examined overnight in 30 male basketball players using a between-groups design [10]. The players completed a performance test battery during the day (i.e., countermovement jump, repeated-sprint test, 5-0-5 agility test) before and after a 40 min fatigue-inducing basketball-specific session. The players were randomly assigned to a control group, who wore loose-fitting garments to bed, or a compression group, who wore lower-body compression garments to bed. Total sleep time was assessed using a wrist activity monitor and sleep quality was assessed using a 5-point Likert scale. There was a small positive effect (*d* = 0.42) of wearing compression garments on subjective sleep quality, but objective total sleep time did not differ between the control group and the compression group.

Two potential limitations to consider when interpreting the results of the study described above [10] include (i) the use of wrist activity monitors to assess sleep and (ii) the use of a between-groups experimental design to examine the impact of compression garments on sleep. Wrist activity monitors tend to underestimate wake duration and do not provide information on the depth (i.e., structure) of sleep [23,24,25]. The gold standard for monitoring sleep is polysomnography (i.e., recordings of the electroencephalogram, electrooculogram, and skeletal muscle electromyogram) because it provides accurate information regarding the duration and structure of sleep (i.e., time spent in light sleep, slow wave sleep, rapid eye movement sleep, arousals) [26,27]. Compression garments may affect the structure of sleep, but this can only be determined if sleep is assessed with polysomnography. In a between-subjects design, participants are exposed to only one condition—i.e., treatment or control. This approach can be used to evaluate the efficacy of a treatment, but differences in the response between individuals can make it hard to detect the effect of the treatment on the outcome of interest. For example, the standard deviation of mean sleep duration was substantially higher in a group of participants who wore compression garments to bed compared to a control group who did not wear compression garments to bed (i.e., 45 min vs. 24 min) [10]. A within-subjects design that includes a rigorous assessment of sleep using polysomnography may be required to determine whether wearing compression garments to bed following a bout of exercise affects sleep.

The aim of the present study was to examine the impact of wearing lower-body compression tights on the quantity and quality of sleep obtained by healthy male adults following a bout of moderate-intensity daytime exercise. The bout of exercise was of sufficient duration and intensity (i.e., 40 min on a cycle ergometer at 70% of age-predicted maximal heart rate; HR) to elicit muscle soreness and pain in this population [28]. It was hypothesized that wearing lower-body compression tights to bed would reduce sleep onset latency, increase total sleep time, reduce wake duration, reduce the number of arousals, and increase the time spent in slow-wave sleep.

## 2. Materials and Methods

### 2.1. Participants

Twelve untrained male volunteers (age = 24.0 ± 1.4 years; body mass index = 24.3 ± 1.4 kg·m^−2^; mean ± standard error of the mean; SEM) gave written, informed consent to participate in the study. The analyses presented in this manuscript are based on secondary data that were collected in a broader study that was primarily designed to examine the impact of a single bout of afternoon exercise on subsequent night-time sleep [29]. Therefore, the study was powered to address the primary research questions rather than the secondary research questions. Two power analyses indicated that the study had relatively low power regarding the secondary research questions. In particular, the study had 39–49% power to detect the potential impact of compression garments on the main variable related to objective sleep quality, i.e., amount of slow-wave sleep. Effect sizes for these power analyses were based on (a) the effect size observed in a previous study that examined the impact of compression garments on subjective sleep quality (*d* = 0.42) [10], and (b) the minimum effect size considered to be practically meaningful (*d* = 0.5, equating to a 10–15% difference in slow-wave sleep). Participants were in good physical and mental health and had no medical, psychiatric, circulatory or sleep disorders as determined by interviews and screening questionnaires. Participants had regular sleep patterns, were non-smokers, were not taking sleep medications, were free from injury, and had not undertaken shift work or flight across more than two time zones in the three months prior to the study. The participants reported a usual sleep duration of 7.6 ± 0.4 h per night and performed 245 ± 52 min of at least moderate-intensity physical activity/exercise per week. The participants were classified as recreationally active (i.e., Tier 1) according to a published framework [30]. The study was conducted in accordance with the Declaration of Helsinki and was approved by the CQUniversity Human Research Ethics Committee.

### 2.2. Study Design

A within-subjects, counterbalanced, randomized design was employed to examine the impact of compression tights on sleep. Participants completed two conditions over three consecutive days and nights in a sleep laboratory: (i) Treatment—wearing compression tights during sleep after exercise, and (ii) Control—not wearing compression tights during sleep after exercise. Participants completed 40 min of moderate-intensity cycling exercise each afternoon and were given a 9 h sleep opportunity each night.

### 2.3. Study Conditions

The study was conducted in a sound-attenuated and temperature-controlled (target temperature = 21–23 °C) accommodation suite at the Appleton Institute for Behavioral Science. Participants were each allocated a private bedroom and bathroom. Light intensity was 350 lux during wake periods (measured 183 cm above floor level with a horizontal angle of gaze) and <0.03 lux during sleep opportunities.

### 2.4. Procedure

In the week prior to the study, participants completed a submaximal graded exercise test to establish the power output for the moderate-intensity exercise sessions and the appropriate size of compression tights. Participants then spent three consecutive days and nights in the accommodation suite to complete the protocol. The protocol began with a 9 h adaptation sleep on night 1 (i.e., 23:00–08:00) to familiarize participants with the equipment for monitoring sleep. On days 2 and 3, participants performed 40 min of moderate exercise on a cycle ergometer in the afternoon (i.e., 14:00–16:00). On nights 2 and 3, participants were provided with a 9 h sleep opportunity (i.e., 23:00–08:00). Polysomnography equipment was applied in the 60 min prior to bed. In the treatment condition, participants wore lower-body compression tights between 22:00 and 08:00 (i.e., participants did not wear compression tights during the exercise session). Thirty minutes after waking (08:30), participants rated their subjective sleepiness and subjective sleep quality, as well as their experience of comfort and pain during the previous night’s sleep.

In the time between exercise sessions and bedtime, participants were permitted to watch movies, listen to music, or read but were not permitted to perform any physical activity or sleep outside the designated opportunities. Participants were provided standardized meals and snacks at the same time each day that were consistent with the macronutrient content of a typical Western diet. Participants were not permitted to consume any caffeine or alcohol during the protocol [31,32]. Participants had access to e-devices during wake periods, but these were removed 30 min prior to bed. Participants were not permitted to leave the accommodation suite during the protocol.

### 2.5. Lower-Body Compression Garment

The lower-body compression garments used in this study were full-length ‘Refresh Recovery Tights’ (MA4419b, 2XU, Melbourne, VIC, Australia). Participants wore an appropriate size of tights according to their height and body mass as per the manufacturer’s guidelines.

### 2.6. Polysomnography

Sleep was monitored using polysomnography (Compumedics Grael, Melbourne, VIC, Australia). A standard montage of electrodes was applied to the scalp and face in the 30 min prior to bed. The montage included three electroencephalograms (C4-M1, F4-M1, O2-M1), two electrooculograms (left outer canthus and right outer canthus), and three electromyograms. An experienced sleep technician continuously monitored the quality of the signals during each sleep opportunity. Sleep records were scored in accordance with established criteria [27] by the same trained technician who was blind to the conditions of the study. The following variables were calculated from each record—(i) sleep onset latency, (ii) total sleep time, (iii) time spent in stage N1, N2, N3 and rapid eye movement (REM) sleep, (iv) wake duration, (v) sleep efficiency, and (vi) number of arousals.

### 2.7. Subjective Ratings of Sleepiness, Sleep Quality, Total Sleep Time, Ease of Falling Asleep, Comfort, Temperature, Pain and Ease of Waking

Subjective sleepiness was measured using the 9-point Karolinska Sleepiness Scale (KSS), where 1 = “extremely alert”, 4 = “fairly alert”, and 9 = “very sleepy, great effort to keep awake, fighting sleep” [33]. Subjective sleep quality was assessed using a 7-point scale, where 1 = “extremely poor”, 4 = “average”, and 7 = “extremely good” and subjective total sleep time was assessed with the question “In your 9 h in bed, how much sleep does it feel like you got?”. Subjective ease of falling asleep, comfort, temperature, pain and ease of waking were assessed using visual analogue scales (VAS). Each VAS consisted of a 100 mm horizontal line anchored with statements at each end. Participants place a vertical mark through a point on the line that corresponds to their experience of the construct of interest. A score from 0 to 100 was obtained for each VAS:

- “How easy was it for you to fall asleep last night?”—anchored with “not easy at all” and “very easy”;
- “How comfortable were you during your sleep last night?”—anchored with “not comfortable at all” and “very comfortable”;
- “How hot did you feel during your sleep last night?”—anchored with “not hot at all” and “very hot”;
- “How cold did you feel during your sleep last night?”—anchored with “not cold at all” and “very cold”;
- “Did you experience any pain during your sleep last night?”—anchored with “no pain at all” and “a lot of pain”;
- “How easy was it for you to wake up this morning?”—anchored with “not easy at all” and “very easy”.

### 2.8. Heart Rate and Perceived Exertion

Heart rate (HR) was measured continuously during exercise using a Polar M400 heart rate monitor and chest strap (M400, Polar Electro; Kempele, Finland). Rating of perceived exertion (RPE) was assessed using the modified 10-point Borg Scale [34].

### 2.9. Submaximal Graded Exercise Test

The submaximal graded exercise test was performed on a stationary cycle ergometer (Wattbike Pro, Wattbike Ltd., Nottingham, UK). Participants completed a 5 min warm up at a self-selected intensity corresponding to an RPE of 5 or below, followed by 2 min of rest. The test began with participants cycling at 55 W at 60 rev·min^−1^; thereafter, power output was increased 15 W every minute by increasing cadence. Participants rated their RPE each minute and the test ended when participants reported an RPE of “very severe” (i.e., 7/10). For each participant, the values for power output and HR obtained for each minute were plotted and the predicted power output corresponding to 70% of age-predicted maximal HR was extrapolated [35]. The mean HR and power output corresponding to 70% of age-predicted maximal HR were 134 ± 1 beats·min^−1^ and 109 ± 12 watts, respectively.

### 2.10. Moderate-Intensity Exercise Session

The moderate-intensity exercise session was performed on a stationary cycle ergometer (Wattbike Pro, Wattbike Trainer; Wattbike Ltd., Nottingham, UK). The session began with a 5 min warm-up at 50% of age-predicted maximal HR, followed by 2 min of rest. Participants then completed 40 min of cycling at 70% of age-predicted maximal HR. A 2 min cool down was performed at the end of the session. HR was monitored continuously to ensure participants maintained a power output corresponding to 70% of age-predicted maximal HR. If HR was below or above the target value, the air resistance setting on the cycle ergometer was adjusted accordingly. RPE was assessed every 5 min during exercise.

### 2.11. Statistical Analyses

Variables were examined for normality using the Shapiro–Wilk’s W statistic. The main effect of condition (i.e., no compression vs. compression) on the objective and subjective variables related to sleep was examined using paired *t*-tests for normally distributed variables and Wilcoxon signed-rank tests for non-normally distributed variables. For all variables, difference scores were calculated by subtracting the control score from the treatment score. Effect sizes and corresponding 95% confidence intervals (CI) were calculated using Hedges’ *g* for paired *t*-tests and by dividing the absolute standardized Z statistic by the square root of the number of pairs for Wilcoxon signed-rank tests (*r*). Hedges’ *g* effect sizes were interpreted as small (0.2), medium (0.5), and large (0.8); for *r*, effect sizes were interpreted as trivial (≤0.10); small (≤0.30); medium (≤0.5); and large > 0.5 [36]. The results are reported as mean ± SEM and were considered significant at *p* < 0.05. All analyses were performed using SPSS (Version 28, IBM, Armonk, NY, USA).

## 3. Results

### 3.1. Moderate-Intensity Exercise Sessions

There was no significant difference between conditions for mean relative HR during exercise (control: 70.3 ± 0.2%; treatment: 70.0 ± 0.3%); mean absolute HR during exercise (control: 134.3 ± 0.7 beats·min^−1^; treatment: 133.8 ± 0.9 beats·min^−1^); mean power output during exercise (control: 103.6 ± 16.0 W; treatment: 102.3 ± 14.8 W); and mean RPE during exericse (control: 4.1 ± 0.4%; treatment: 3.9 ± 0.4%) (Table 1). There was no significant difference between the target HR (i.e., 70% of age-predicted maximal HR) and mean HR during the moderate-intensity exercise session in the control condition (*t*_11_ = 1.515, *p* = 0.158, *g* = 0.422, 95% CI = −0.160 to 0.987) or in the treatment condition (*t*_11_ = −0.147, *p* = 0.886, *g* = −0.041, 95% CI = −0.587 to 0.506).

### 3.2. Sleep

**Figure 1:**
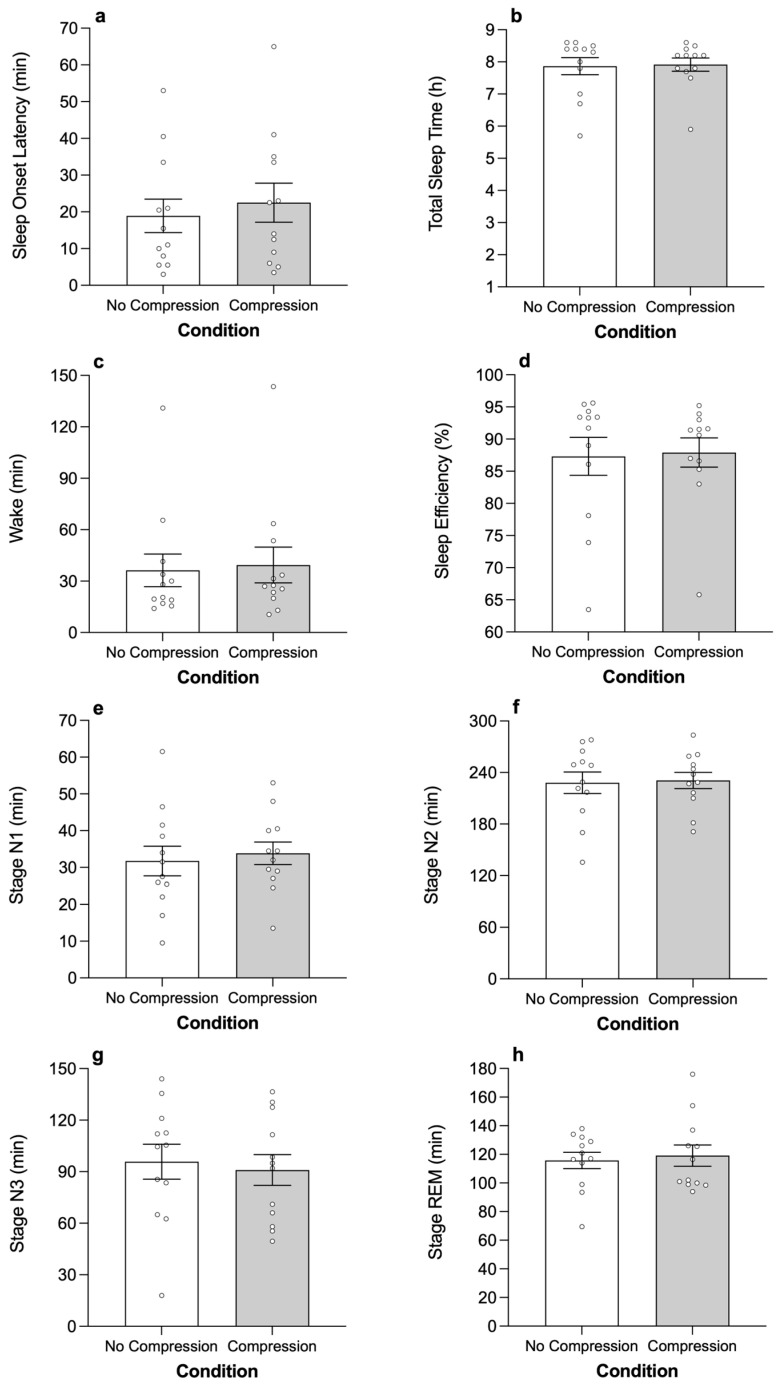
The impact of wearing lower-body compression tights to bed on sleep onset latency (**a**); total sleep time (**b**); wake duration (**c**); sleep efficiency (**d**); stage N1 sleep (**e**); stage N2 sleep (**f**); stage N3 sleep (**g**); and stage rapid eye movement (REM) sleep (**h**). Columns and errors bars are mean ± standard error of the mean (SEM) and open circles are individual cases.

There was no significant difference between conditions for sleep onset latency, total sleep time, wake duration, sleep efficiency (Table 2; Figure 1) or the number of arousals during sleep (control: 119 ± 14; treatment: 116 ± 9). The duration of time spent in each stage of sleep was not significantly different between conditions (Table 2; Figure 1). There was also no significant difference in the percentage of time spent in stage N1 sleep (*p* = 0.656), stage N2 sleep (*p* = 0.945), stage N3 sleep (*p* = 0.256) or Stage REM sleep (*p* = 0.679) between conditions. Differences in sleep variables between the adaptation night and the control and treatment nights and the impact of order on the sleep variables (i.e., night 1 vs. night 2) are reported in the Supplementary Material (Tables S1a and S1b).

### 3.3. Subjective Ratings

**Figure 2:**
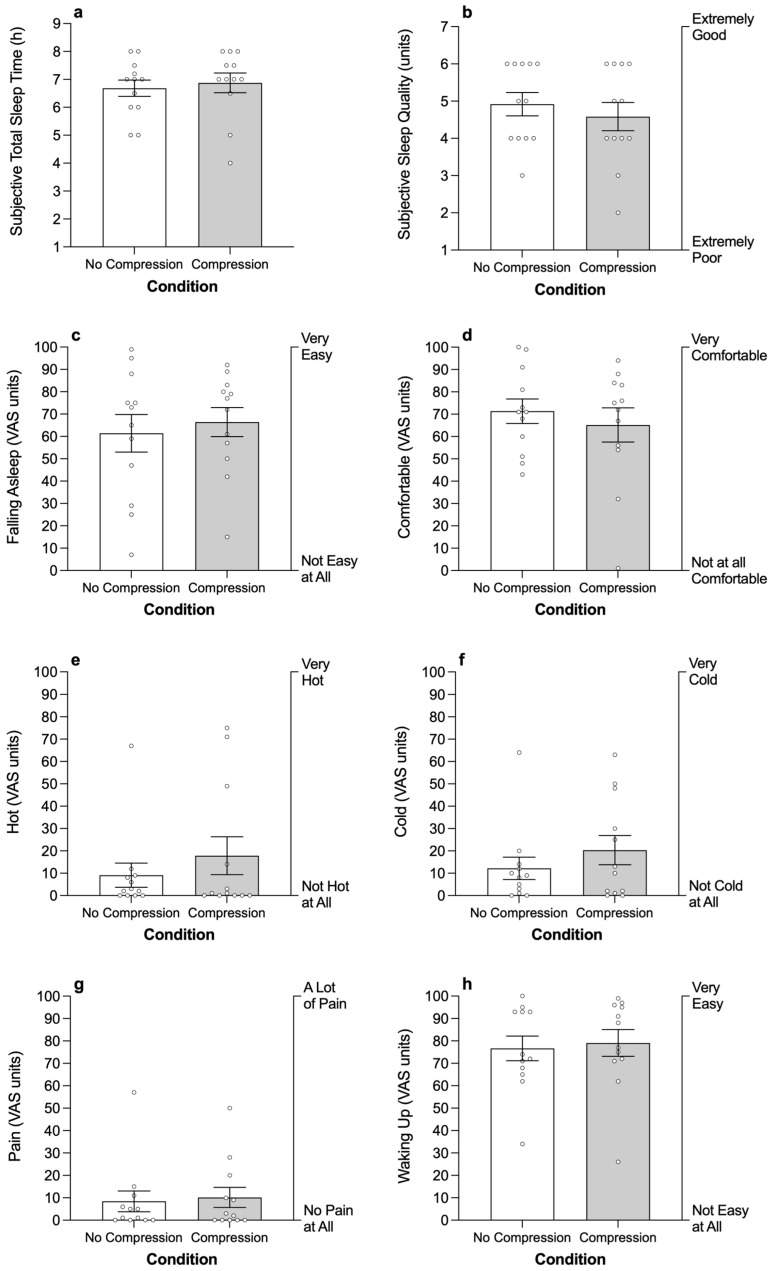
The impact of wearing lower-body compression garments to bed on self-reported total sleep time (**a**); sleep quality (**b**); ease of falling asleep (**c**); comfort during sleep (**d**); temperature during sleep (feeling hot) (**e**); temperature during sleep (feeling cold) (**f**); pain during sleep (**g**); and ease of waking in the morning (**h**). Columns and errors bars are mean ± standard error of the mean and open circles are individual cases.

There was no significant difference between conditions for subjective sleepiness (control: 3.5 ± 0.2 AU; treatment: 3.9 ± 0.4 AU), subjective sleep quality, or subjective total sleep time (Table 3; Figure 2). There was no significant difference between condtions for the subjective ratings of comfort, ease of falling asleep, temperature, pain during sleep, or ease of waking up (Table 3; Figure 2).

## 4. Discussion

The aim of the present study was to examine the impact of wearing lower-body compression tights to bed on sleep quantity and quality following a bout of moderate-intensity daytime exercise. It was hypothesized that wearing lower-body compression tights to bed would reduce sleep onset latency, increase total sleep time, reduce wake after sleep onset, reduce the number of arousals, and increase the time spent in slow wave sleep. However, wearing compression tights to bed had no significant effect on any of the objective measures of sleep quantity or quality. In addition, wearing compression tights to bed had no significant effect on subjective sleep quality or perceptions of pain, comfort, or temperature during sleep. The study had relatively low power to detect these potential effects, but all the effect sizes were negligible, small, and in some cases in the opposite direction to that hypothesized, so it is unlikely that including a larger number of participants would have altered the outcome. Wearing lower-body compression tights to bed after a single bout of moderate-intensity daytime exercise may not improve sleep, but the results of the present study also indicate that wearing lower-body compression tights to bed does not impair the quantity or quality of sleep obtained.

In a previous study in male basketball players—in which wrist activity monitors were used to assess sleep—wearing lower-body compression tights to bed following a bout of fatiguing exercise had no significant effect on total sleep time compared to not wearing compression garments to bed [10]. This finding was supported in the present study—participants obtained ~7.9 h of sleep with and without wearing compression tights to bed. In addition, the use of polysomnography in the present study provided information on the structure of sleep that cannot be obtained when using wrist activity monitors [26,27]. In both conditions, participants spent a similar amount of time in light sleep, deep sleep and REM sleep and the sleep architecture observed for sleep episodes in both conditions was comparable to that of healthy male adults [37]. It is important to note that participants in the present study were relatively good sleepers. Consequently, there may have been little room for improvement in sleep quantity or quality (i.e., ceiling effect) when wearing compression tights to bed [38]. Assessing the effects of compression tights on sleep in poor sleepers may yield different results and may be of interest in future studies.

Wearing lower-body compression garments following a bout of exercise can reduce perceptions of muscle soreness [7,8,9,10] and fatigue [9,10]. Pain and discomfort can delay sleep onset latency and increase the likelihood of waking during the night [18,19]. One avenue through which lower-body compression garments are thought to facilitate sleep following a bout of exercise is through a reduction in perceived muscle soreness or pain [10]. In the present study, perceptions of pain, comfort and sleep quality were not different between the compression condition and the control condition. There may be several explanations for this finding. For example, it is possible that compression garments do not reduce perceptions of muscle pain, muscle soreness, or fatigue following a bout of moderate-intensity exercise. Indeed, in some studies, compression garments have no effect on post-exercise perceptions of muscle soreness [9,39,40,41] or muscle pain [42,43,44]. However, it is also possible that the exercise stimulus employed in the present study—i.e., 40 min of primarily concentric exercise at 70% of age-precited maximal HR—was not of sufficient intensity to elicit symptoms of muscle soreness or pain. Muscle soreness is typically associated with high-force muscular work and is precipitated by eccentric actions [45]. In studies where a bout of eccentric exercise has been employed, self-reported muscle soreness is lower in participants wearing compression garments compared to participants not wearing compression garments [46]. It seems reasonable to suggest that if there are benefits of wearing compression garments on sleep, these benefits may only be apparent following a bout of exercise that is likely to induce muscle soreness (i.e., eccentric exercise).

Perceptions of muscle soreness typically begin 6–12 h after exercise, before reaching peak levels of intensity 24–72 h after exercise [45,47]. If the mechanism through which compression garments facilitate sleep involves reduced perceptions of muscle soreness, then improvements in sleep may only emerge when muscle soreness is at its peak (i.e., 2–3 nights after exercise). In the present study, sleep was assessed ~7 h after exercise. This time frame of assessment most likely not include the period of peak muscle soreness in our participants. Therefore, consecutive nights of monitoring may be required in future studies in which the purpose is to examine potential mechanisms through which compression tights alter sleep quantity and quality.

There are some limitations that should be considered when interpreting the results of the present study. First, data were collected with a small group of untrained, physically active males. The results may not be generalizable to females, sedentary individuals, or highly trained individuals. In addition, other factors (e.g., age, chronotype, habitual sleep duration, etc.) may be of interest in future studies in which the purpose is to examine the impact of wearing compression tights to bed on sleep. Second, the stimulus employed in the present study consisted of 40 min of moderate-intensity aerobic exercise on a cycle ergometer. Wearing lower-body compression tights to bed may be beneficial for sleep when the exercise stimulus is longer in duration (i.e., >40 min), higher in intensity (i.e., >70% age-predicted maximal HR) or different in modality (e.g., running or resistance exercise). Third, there is some evidence to indicate that chronic use of compression garments provides small to moderate effects for reduced perceptions of muscle soreness [48]. In the present study, we examined the acute impact of wearing compression tights to bed on sleep and thus we cannot exclude the possibility that long-term use of compression tights may be beneficial to sleep. Finally, participants were provided with fixed sleep opportunities (i.e., 22:30–08:00) to ensure time in bed and exposure to light were identical between conditions. However, fixed sleep and light schedules may not reflect habitual sleep timing and may compromise ecological validity [49].

## 5. Conclusions

Wearing lower-body compression tights to bed following 40 min of moderate-intensity exercise (70% of age-predicted maximal HR) had no effect on objective or subjective measures of sleep quantity or quality in untrained, healthy male adults. The results of the present study indicate that wearing lower-body compression tights to bed after a single bout of moderate-intensity daytime exercise may not improve sleep but neither does it interfere with the quantity or quality of sleep obtained.

## Figures and Tables

**Table 1:** Statistical results of the main effect of condition on exercise variables.

Variable	Statistic	*p* Value	Effect Size (95% CI)
Relative HR (%)	*Z* = −0.764	0.445	−0.226 *^r^* (−0.751 to 0.454)
Absolute HR (beats·min^−1^)	*t*_11_ = −0.939	0.368	−0.262 *^h^* (−0.813 to 0.301)
Power output (W)	*t*_11_ = −0.485	0.637	−0.135 *^h^* (−0.681 to 0.417)
RPE (units)	*t*_11_ = −0.458	0.656	−0.128 *^h^* (−0.674 to 0.424)

HR, heart rate; RPE, rating of perceived exertion; CI, confidence interval; *^r^*, *r* effect size; *^h^*, Hedges’ *g* effect size.

**Table 2:** Statistical results of the main effect of condition on objective sleep variables.

Variable	Statistic	*p* Value	Effect Size (95% CI)
Sleep onset latency (min)	*t*_11_ = 0.582	0.572	0.162 *^h^* (−0.392 to 0.709)
Total sleep time (h)	*Z* = 0.059	0.953	0.020 *^r^* (−0.750 to 0.647)
Wake (min)	*Z* = 0.941	0.347	0.124 *^r^* (−0.476 to 0.638)
Sleep efficiency (%)	*Z* = 0.314	0.754	0.091 *^r^* (−0.456 to 0.681)
Arousals (count)	*t*_11_ = −0.293	0.775	−0.082 *^h^* (−0.627 to 0.467)
Stage N1 sleep (min)	*t*_11_ = 0.534	0.604	0.149 *^h^* (−0.404 to 0.695)
Stage N2 sleep (min)	*t*_11_ = 0.188	0.854	0.052 *^h^* (−0.496 to 0.598)
Stage N3 sleep (min)	*t*_11_ = −0.927	0.374	−0.258 *^h^* (−0.809 to 0.304)
Stage REM sleep (min)	*Z* = 0.471	0.638	0.136 *^r^* (−0.413 to 0.690)

REM, rapid eye movement; CI, confidence interval; *^h^*, Hedges’ *g* effect size; *^r^*, *r* effect size.

**Table 3:** Statistical results of main effect of condition on subjective sleep variables.

Variable	Statistic	*p* Value	Effect Size (95% CI)
Subjective sleepiness (AU)	*t*_11_ = 1.101	0.295	0.307 *^h^* (−0.261 to 0.861)
Subjective sleep quality (AU)	*Z* = −0.600	0.549	−0.200 *^r^* (−0.801 to 0.502)
Subjective total sleep time (h)	*Z* = 0.448	0.654	0.135 *^r^* (−0.473 to 0.693)
VAS falling asleep (AU)	*t*_11_ = 0.466	0.664	0.124 *^h^* (−0.427 to 0.670)
VAS comfort (AU)	*t*_11_ = −0.620	0.548	−0.173 *^h^* (−0.720 to 0.382)
VAS hot (AU)	*Z* = 1.053	0.292	0.371 *^r^* (−0.457 to 0.835)
VAS cold (AU)	*Z* = 0.845	0.398	0.254 *^r^* (−0.347 to 0.797)
VAS pain (AU)	*Z* = 0.204	0.838	0.065 *^r^* (−0.598 to 0.648)
VAS waking up (AU)	*Z* = 0.864	0.387	0.249 *^r^* (−0.320 to 0.777)

AU, arbitrary units; VAS, visual analogue scale; CI, confidence interval; *^h^*, Hedges’ *g* effect size; *^r^*, *r* effect size.

## Data Availability

Data supporting the reported results are available from the corresponding author on reasonable request.

## Supplementary Materials

The following supporting information can be downloaded at: https://www.mdpi.com/article/10.3390/s26051625/s1, Table S1a: Statistical results of the main effect of order on sleep and exercise variables; Table S1b: Comparison of sleep variables between the adaptation night, control night (no compression) and the treatment night (compression).

## Abbreviations

The following abbreviations are used in this manuscript:

AU	Arbitrary units
CI	Confidence interval
HR	Heart rate
KSS	Karolinska Sleepiness Scale
REM	Rapid eye movement
REV	Revolutions
RPE	Rating of perceived exertion
SEM	Standard error of the mean
VAS	Visual analogue scale
