# Supplementary material for: Wearing Lower-Body Compression Tights to Bed After Cycling Exercise Does Not Affect Subsequent Sleep in Healthy Male Adults
Source: Sensors (Basel). 2026 Mar 5;26(5):1625. doi: 10.3390/s26051625 (PMC12987341; doi:10.3390/s26051625)
Supplement: Supplementary file 1 [file sensors-26-01625-s001.zip › sensors-4112238-supplementary.pdf]

SUPPLEMENTARY MATERIAL

Wearing lower-body compression tights to bed after cycling exercise does not affect subsequent sleep in healthy male adults

Charli Sargent[1,2*], Shona L. Halson[3], Matthew Morrison[4], Carissa L. Gardiner[3], Dean J. Miller[1,2], Bree Elliott[1], Katrina Nguyen[1], James R. Broatch[5], Jonathon Weakley[3], Gregory D. Roach[1,2]

Table S1a. Statistical results of the main effect of order on sleep and exercise variables.

Variable	Statistic	p Value
Sleep onset latency (min)	$Z = -0.314$	.753
Total sleep time (h)	$Z = 0.275$	.784
Wake (min)	$Z = 0.118$	.906
Sleep efficiency (%)	$Z = 0.314$	.754
Arousals (count)	$t_{11} = -1.562$	.147
Stage N1 sleep (min)	$t_{11} = -0.644$	.532
Stage N2 sleep (min)	$t_{11} = -0.380$	.711
Stage N3 sleep (min)	$t_{11} = -0.403$	.695
Stage REM sleep (min)	$t_{11} = -0.665$	.520
Subjective sleepiness (AU)	$t_{11} = -0.209$	.838
Subjective sleep quality (AU)	$Z = 0.480$	.631
Subjective total sleep time (h)	$Z = -0.179$	.858
VAS falling asleep (AU)	$t_{11} = -0.787$	.448
VAS comfort (AU)	$t_{11} = -0.869$	.403
VAS hot (AU)	$Z = -1.474$	.141
VAS cold (AU)	$Z = -1.913$	.056
VAS pain (AU)	$Z = -1.785$	.074
VAS waking up (AU)	$Z = -1.257$	.209

The effect of order (i.e., night 1 vs. night 2) on the variables related to sleep and exercise were examined using paired t-tests for normally distributed variables and Wilcoxon signed-rank tests for non-normally distributed variables. REM, rapid eye movement; AU, arbitrary units; VAS, visual analogue scale.

Table S1b. Comparison of sleep variables between the adaptation night, control night (no compression) and the treatment night (compression).

Variable	Statistic	p Value
Sleep onset latency (min)	$F_{2,22} = 0.930$	.373
Total sleep time (h)	$\chi^2 = 2.00$	.368
Wake (min)	$\chi^2 = 0.667$	.717
Sleep efficiency (%)	$\chi^2 = 2.00$	.368
Arousals (count)	$F_{2,22} = 0.698$	.508
Stage N1 sleep (min)	$F_{2,22} = 1.614$	.222
Stage N2 sleep (min)	$F_{2,22} = 0.676$	.519
Stage N3 sleep (min)	$F_{2,22} = 0.323$	.728
Stage REM sleep (min)	$\chi^2 = 3.106$	.212
Subjective sleep quality (AU)	$\chi^2 = 1.421$	.491
Subjective total sleep time (h)	$\chi^2 = 1.442$	.486

Differences in sleep variables between nights (i.e., adaptation, no compression, compression) were examined using one-way repeated measures analysis of variance for normally distributed variables and the Friedman test for non-normally distributed variables. REM, rapid eye movement; AU, arbitrary units.
